# Preclinical Models for Neuroblastoma: Establishing a Baseline for Treatment

**DOI:** 10.1371/journal.pone.0019133

**Published:** 2011-04-29

**Authors:** Tal Teitz, Jennifer J. Stanke, Sara Federico, Cori L. Bradley, Rachel Brennan, Jiakun Zhang, Melissa D. Johnson, Jan Sedlacik, Madoka Inoue, Ziwei M. Zhang, Sharon Frase, Jerold E. Rehg, Claudia M. Hillenbrand, David Finkelstein, Christopher Calabrese, Michael A. Dyer, Jill M. Lahti

**Affiliations:** 1 Department of Tumor Cell Biology, St. Jude Children's Research Hospital, Memphis, Tennessee, United States of America; 2 Department of Developmental Neurobiology, St. Jude Children's Research Hospital, Memphis, Tennessee, United States of America; 3 Department of Hematology/Oncology, St. Jude Children's Research Hospital, Memphis, Tennessee, United States of America; 4 Animal Imaging Center, St. Jude Children's Research Hospital, Memphis, Tennessee, United States of America; 5 Department of Radiological Sciences, St. Jude Children's Research Hospital, Memphis, Tennessee, United States of America; 6 Cell and Tissue Imaging, St. Jude Children's Research Hospital, Memphis, Tennessee, United States of America; 7 Department of Pathology, St. Jude Children's Research Hospital, Memphis, Tennessee, United States of America; 8 Information Sciences, St. Jude Children's Research Hospital, Memphis, Tennessee, United States of America; 9 Department of Ophthalmology, University of Tennessee Health Science Center, Memphis, Tennessee, United States of America; 10 Howard Hughes Medical Institute, Chevy Chase, Maryland, United States of America; 11 Department of Molecular Sciences, University of Tennessee Health Science Center, Memphis, Tennessee, United States of America; Virginia Commonwealth University, United States of America

## Abstract

**Background:**

Preclinical models of pediatric cancers are essential for testing new chemotherapeutic combinations for clinical trials. The most widely used genetic model for preclinical testing of neuroblastoma is the TH-MYCN mouse. This neuroblastoma-prone mouse recapitulates many of the features of human neuroblastoma. Limitations of this model include the low frequency of bone marrow metastasis, the lack of information on whether the gene expression patterns in this system parallels human neuroblastomas, the relatively slow rate of tumor formation and variability in tumor penetrance on different genetic backgrounds. As an alternative, preclinical studies are frequently performed using human cell lines xenografted into immunocompromised mice, either as flank implant or orthtotopically. Drawbacks of this system include the use of cell lines that have been in culture for years, the inappropriate microenvironment of the flank or difficult, time consuming surgery for orthotopic transplants and the absence of an intact immune system.

**Principal Findings:**

Here we characterize and optimize both systems to increase their utility for preclinical studies. We show that TH-MYCN mice develop tumors in the paraspinal ganglia, but not in the adrenal, with cellular and gene expression patterns similar to human NB. In addition, we present a new ultrasound guided, minimally invasive orthotopic xenograft method. This injection technique is rapid, provides accurate targeting of the injected cells and leads to efficient engraftment. We also demonstrate that tumors can be detected, monitored and quantified prior to visualization using ultrasound, MRI and bioluminescence. Finally we develop and test a “standard of care” chemotherapy regimen. This protocol, which is based on current treatments for neuroblastoma, provides a baseline for comparison of new therapeutic agents.

**Significance:**

The studies suggest that use of both the TH-NMYC model of neuroblastoma and the orthotopic xenograft model provide the optimal combination for testing new chemotherapies for this devastating childhood cancer.

## Introduction

Neuroblastoma (NB) is responsible for 15% of all childhood cancer deaths and is the most common cancer diagnosed during the first year of life [1]. NB arises in the developing sympathetic nervous system, in precursor cells thought to be derived from the neural crest tissues [2]. The tumors appear in the adrenal medulla or along the paraspinal ganglia in the abdomen, chest, pelvis or neck [3], [4].

A new International Neuroblastoma Risk Group (INRG) classification system of the disease divides the patients to 16 risk groups from the lowest risk group with localized tumor that can be removed by surgery and has a greater than 95% survival rate, to the highest risk group that presents with metastasis to bone marrow and bone and currently has only 40 to 50% survival rate [5], [6]. A unique patient group is the 4S which usually occurs in infants less then one year of age and has a favorable prognosis with a greater than 90% survival rate. Although the tumors in the 4S group develop very early, they undergo spontaneous regression [7]. Full regression is also seen in some of the stage 1 tumors with localized disease [8].

Amplification of N-MYC (>10 copies per cell) occurs in about 30% of NB human patients and is strongly correlated with advanced disease stage and poor outcome [9]–[11]. Several studies show that MYC proteins can act as master transcriptional factors to activate or repress a wide variety of genes [12], [13]. In addition the MYC family proteins including MYCN can influence expression of genes through deregulation of microRNAs [14]–[17]. Importantly, high expression of N-myc is sufficient to induce neuroblastoma tumor formation in transgenic mice [18].

The TH-MYCN transgenic mouse model, in which N-myc expression is driven by a 4.5 Kb promoter of the rat tyrosine hydroxylase gene (TH) which is expressed specifically in neural crest lineage cells, is now a widely used murine model of NB [18]. Tumor penetrance in this transgenic NB model is strain dependent [18]. NB tumors arise in the TH-MYCN transgenic model at high frequency on the 129SvJ background, with ∼33% of the hemizygous transgenic mice and 100% of the homozygous mice developing tumors. The reason for the high tumor frequency on the 129SvJ background compared to other murine genetic backgrounds, such as the BL6 strain which has only 5% tumor occurrence, is still not clear but is attributed to strain-specific modifiers not identified yet.

Overexpression of N-MYC in the mouse peripheral neural crest of the TH-MYCN mice gives rise to NB tumors that recapitulate many of the histological and pathological aspects of human NB [18]–[20]. In addition, genome-wide array CGH analysis of the murine tumors identified distinct genomic aberrations that share some similarity with those in human tumors [21]. Cell lines derived from the mouse tumors display many of the molecular characteristics of human NB [22] although the tumors rarely exhibit loss of the mouse gene region that is syntenic with chromosome 1p36 or metastasize to distant organs such as bone marrow [21], [23].

Several recent studies have utilized the TH-MYCN transgenic model to understand the *in vivo* functions of additional genes involved in NB pathogenesis such as p53 and Mdm2 [24], [25]. Importantly, this murine model has been very useful in assessing the clinical efficacy of potential new drugs for NB like the angiogenesis inhibitor HPMA copolymer-TNP-470 conjugate (caplostatin) [24] and the ornithine decaroboxylase inhibitor α-difluoromethylornithine [26].

In this study, we provide additional characterization of the TH-MYCN mouse model using multiple diagnostic imaging methodologies and gene profiling. In addition, we present a new approach for generating orthotopic xenografts of neuroblastoma using ultrasound guided injections of neuroblastoma cells into the adrenal or para-adrenal space, where 40% of human NB tumors arise [3]. To demonstrate the utility of this new orthotopic xenograft method for neuroblastoma and provide a clinically relevant benchmark for future preclinical studies, we tested a chemotherapeutic regimen that is similar to the current standard of care for induction chemotherapy for neuroblastoma in the xenograft model. These data set the stage for future generation of orthotopic xenografts of primary human neuroblastoma and for testing new agents for this devastating childhood cancer.

## Results

### Characterization of the cellular features of TH-MYCN neuroblastoma

**Figure 1:**
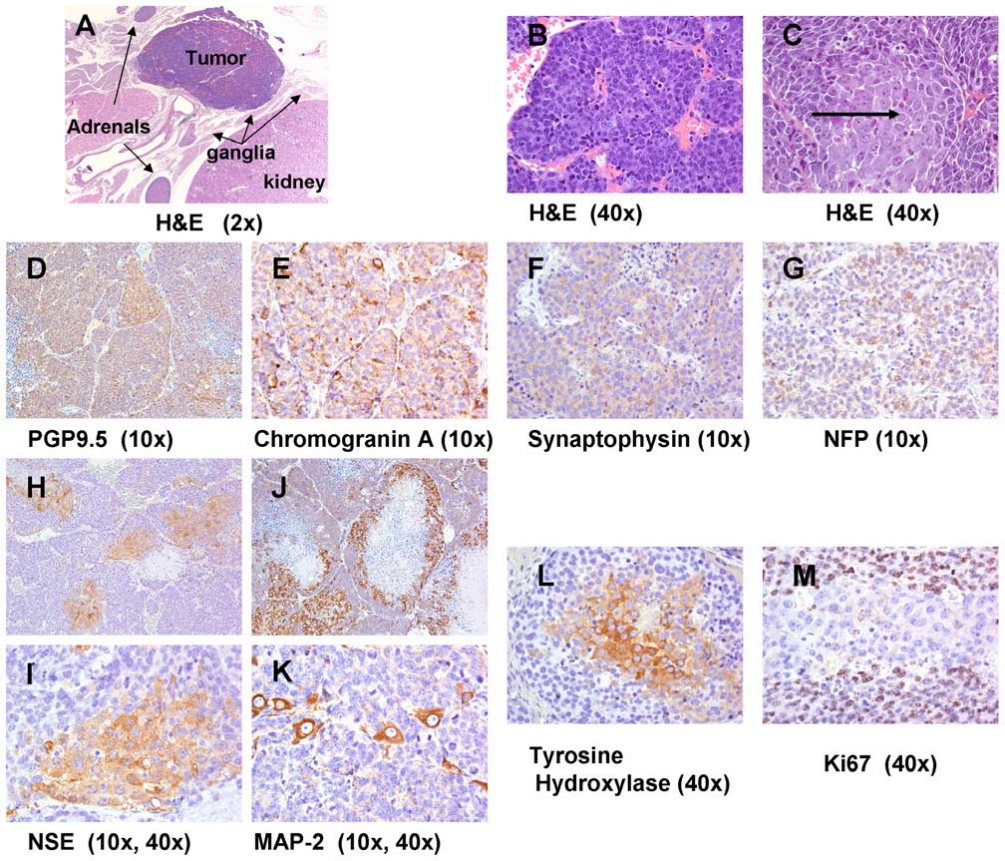
Histology and immunohistochemistry of mouse TH-MYCN tumors. Tumors that arose along the paraverterbral ganglia predominately exhibited (**A**), a solid cord (**B**) and molding/pavement morphologies (**C**) although areas of cells with a ganglion-like appearance were dispersed throughout the tumors (see arrow). The tumors stained homogenously for the neuronal markers- PGP9.5 (**D**), chromogranin A (**E**), synaptophsin (**F**) and neurofilament protein (**G**). However, only the ganglion-like cells within the tumor stained strongly positive for the neuronal markers - neuron specific enolase (**H, I**), microtubule associated protein 2 (**J, K**), Tyrosine Hydroxylase (**L**). These ganglion-like cells were negative for the proliferation marker Ki67 (**M**).

To obtain NB tumors from the TH- MYCN model, hemizygous TH-MYCN transgenic mice were bred onto the 129SvJ background. Tumors arose on this background at a frequency of ∼33%. Tumors could first be detected visually in the abdomen of the transgenic mice at 9–13 weeks of age. Earlier tumors could be routinely identified by imaging (MRI and ultrasound) at week 6(see below). The TH-MYCN mouse tumors appeared to arise in the ganglion cells, in line with the sympathetic ganglion chain (Figure 1A.). We characterized these tumors histologically by Hematoxylin and Eosin (H&E) staining (Figure 1 and Figure S1), by electron microscopy (Figure S2) and by immunohistochemistry (IHC) staining with neuronal markers (Figure 1). Ki67 staining was also employed to evaluate proliferation (Figure 1M). H&E and IHC staining revealed substantial heterogeneity within the primary tumors and the presence of microscopic (<1 mm^3^) metastatic lesions in the lungs (100% of the animals), ovaries (44%), abdominal lymph nodes (32%) and bone marrow (5%) of 3–5 months old mice. The majority of the H&E stained tumor cells were variably sized, blue cells, with generally prominent nucleoli and distinct nuclear membranes that grew in solid sheets, cords and molded pavement patterns (Figure 1B and Figure S1A–D). However, there were also regions of much larger cells that had a more ganglion-like appearance in the primary mouse tumors (Figure 1C and Figure S1E,F).

Analysis of the developing primary TH-MYCN tumors (mouse ages 9–13 weeks) by immunohistochemistry revealed that the mouse tumors stained homogenously for the neuronal markers: protein gene-product 9.5 (PGP9.5), chromogranin A, synapthopysin and neurofilament protein (NFP) (Figure 1D–G). However, there was heterogeneity in the staining patterns with other neuronal markers including neuron specific enolase (NSE), microtubule-associated protein 2 (MAP-2) and tyrosine hydroxylase (TH), a marker of more differentiated cells. These proteins were strongly expressed only in areas of ganglion-like cells within the tumors (Figure 1H–L). The regions of the tumors that stained positive for the markers NSE, MAP-2 and TH were also the only areas that were not undergoing proliferation as judged by the absence of Ki67 staining (Figure 1M). In contrast, the majority of the cells in the tumors stained positive for Ki67, indicating potential proliferation, but failed to express the markers NSE, TH and MAP-2 (Figure 1H–L). Numerous mitotic figures were also present in these areas while the areas surrounded by the more differentiated ganglion-like cells exhibited an absence of mitotic figures and often surrounded by necrotic and apoptotic cells (Figure 1H, J). Electron microscopy of the TH-MYCN tumors and human tumors revealed features consistent with neuronal differentiation including dense core neurosecretory vesicles, synaptic junctions and processes typical of neuronal cells (Figure S2). There were also areas of lipids dispersed throughout the tumors.

Overall, these data are consistent with previous characterization of the TH-MYCN neuroblastomas and they suggest that the mouse neuroblastomas most closely resembled a class of aggressive human neuroblastoma tumors classified as large cell neuroblastoma [27]–[29]. This subgroup of tumors is composed of cells that are markedly larger than other NB subgroups and have unique, sharply outlined nuclear membranes and 1–4 prominent nucleoli. Tumor with this morphology are found within the undifferentiated and poorly differentiated classes of NB and are seen most often in cases with MYCN amplified tumors.

### Molecular profiling of mouse neuroblastomas

To extend these findings, we isolated RNA from 18 samples of hemizygous abdominal TH-MYCN tumors and performed Affymetrix gene expression array analysis. These tumors which were representative of full grown progressive tumors, weighing 1.5 gr+/−0.75 gr, were harvested from 12 females and 6 males 8–13 week old mice. All eighteen samples clustered tightly on principle component analysis (PCA) analysis (data not shown). Human NB data were obtained from three previously published neuroblastoma studies using the Affymetrix U133 v2 chip and combined to give a total of 125 gene expression arrays (Figure S3) for comparison. The data was then examined by PCA and collated with meta data describing each tumor (Figure S3). The 125 arrays were adjusted by batch correction method included in Partek Genomics Suite 6.4. Data from Affymetrix human U133 v2 arrays and mouse Affymetrix 430 v2 arrays were processed using the RMA algorithm. This data was then used to examine data quality by PCA for each species separately. The corrections described above removed much of the variability across the human array data from the different studies (Figure S3).

Next were removed genes that did not have documented orthologs, between humans and mice, using the Affymetrix's ortholog mapping document derived from National Center for Biotechnology Information's (NCBI) Homologene database. The probesets corresponding to 15646 unique human and mouse unigene pairs were retained. Each subset of the original data from mouse and human data sets was then converted to quartiles to further normalize the data and to concentrate on large differences between the species. Once quartiled, the data within each unigene id was averaged and the resulting means were compared across species.

The batch corrected set of human neuroblastomas and the mouse N-MYC tumors unigene pairs were then assigned grades (A+ - D) based on the performance of probesets that were designed to measure the same transcript in the same array. The purpose of this grading was to distinguish consistent measurements from variable ones. The quartile range across all observations within a given unigene and species represents technical and biological variability but does not influence grade. However, the range of probeset signals within a unigene does influence the grade. If the range for all probesets for a particular unigene within a species was less than or equal to 1 quartile it was given an A+ grade. A total of 424 A+ unigene pairs (2.7% of the total unigenes, 424/15646 unigenes) from all stages and MYCN status exhibited expression differences of one or more quartiles between human and mouse and were termed differential (Table S1). Gene ontology, using DAVID's GO analysis [30] showed statistically significant enrichment for genes in the immune response among the 424 differential genes (Table S1). 125 of these genes showed differences in expression of 2 quartiles or more, up or down, between the two species across all disease stages and MYCN status (Table S2). When we compared the expression of the 424 differential unigenes to their expression in normal adrenal tissue (A+ only) of the appropriate species and subtracted these species differences there were 18 unigenes that were differentially expressed between human tumors and mouse tumors (Table S3). These 18 unigenes are presented in Table S3. Among them we find breast cancer 2 early onset gene 2 (BRCA2) which was expressed at significantly higher levels in the mouse tumors.

When we compared the TH-MYCN mouse tumor microarray expression data to the human NB patient data (A+ quality data only as described above) classified by disease stage and restricted to MYCN amplified patients, we found high similarity between stages 3 and 4 of human disease and the mouse model (see Table 1). Interestingly, there were no genes that had a statistically different level of expression in the stage 4 N-MYC amplified human tumors versus mouse tumors and only 53 genes were differentially expressed between mouse and human stage 3 MYCN–amplified samples. Taken together the histological analysis and the molecular analysis suggest that the TH-MYCN mouse model most closely resembles stages 3 and 4 N-MYC amplified human neuroblastoma.

**Table 1:** Differential gene expression between MYCN amplified human neuroblastoma by disease stages and mouse TH-MYCN tumors.

Human Stage(N-MYC amplified)	Number of differences from mouse TH-MYCN tumors
1	135
2	231
3	53
4	0

### Ultrasound guided injection of neuroblastoma cells in mice

While the TH-MYCN tumors had molecular and cellular features of Stage 4 NMYC amplified human neuroblastomas, one major difference was the relatively low rate of distant metastasis in these mice. It is possible that orthotopic xenografts of primary human neuroblastomas or cell lines would more efficiently metastasize than the tumor cells in the TH-MYCN mouse model. Most previous studies focused on xenografts of neuroblastoma have involved flank engraftment or engraftment to the kidney capsule or more rarely engaftment into the adrenal using surgical procedures. However, the microenvironment of the flank is very different from that of the normal site of neuroblastoma initiation and the surgical procedure is relatively slow and can be associated with morbidity. In order to establish a minimally invasive, high throughput method for efficient introduction of neuroblastoma cell lines and primary human tumor cells into the mouse we developed an ultrasound guided technique to inject cells directly into the adrenal or the para-adrenal space. These two areas were chosen because a high percentage of human tumors (40%) are found in these areas [4]. The advantage of this technique is that it is relatively rapid (10–12 mice can be injected per hour), it leads to efficient engraftment of neuroblastoma cell lines (>80%) and there is no morbidity associated with the technique (see Materials and Methods).

Five human NB cell lines were chosen to demonstrate the feasibility and utility of this approach - NB5, NB7, NB1691 [23], [32], [31], SKNAS (ATCC CRL-2137) and SKNSH (ATCC HTB-11). These cell lines were selected because they differ in N-myc amplification, caspase-8 expression, p53 mutation status and 1p36 LOH [23], [32]. The cells were labeled with luciferase by stable transfection with a retrovirus expressing the luciferase gene. Luciferase expression was tested and quantified in the Xenogen Imager before injecting the cells into the mice. Single suspension cells were mixed with matrigel in a total volume of 10 µl and implanted into the para-adrenal area, between the adrenal and the kidney or into the adrenal medulla itself. Injections were done with the aid of an ultrasound-guided catheter and needle (See Materials and Methods). Tumor formation and growth was monitored for up to 24 weeks.

**Figure 2:**
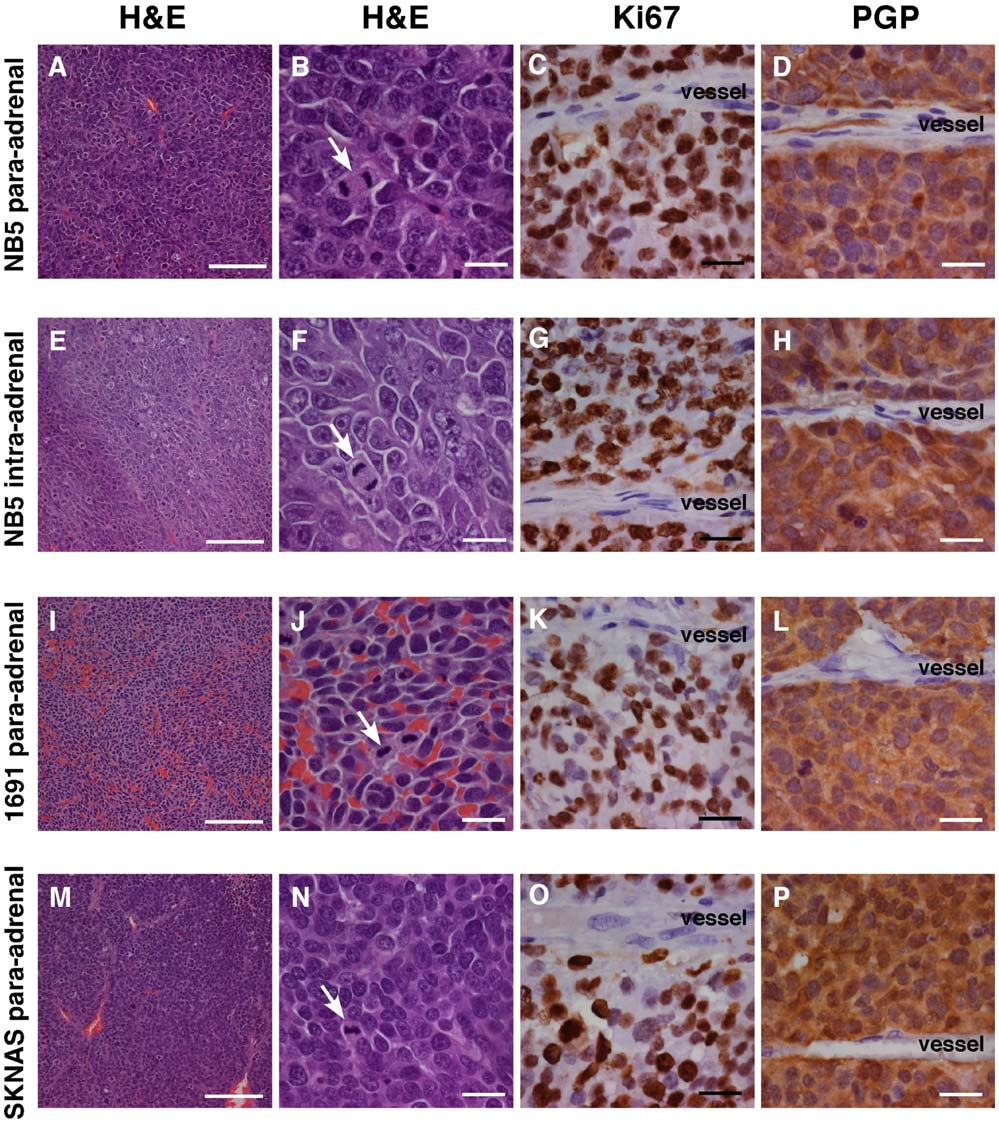
Histology and immunohistochemical analysis of orthotopic neuroblastoma xenografts. NB5 xenografts transplanted into the para-adrenal region (**A–D**) or into the adrenal gland (**E–H**) stained with hematoxylin and eosin (**A, B**), the proliferation marker Ki67 (**C,G**) or PGP9.5 (**D,H**). NB1691 (**I–L**) and SKNAS xenografts (**M-0**) transplanted to the paradrenal region stained with hematoxylin and eosin (**I, J, M N**), Ki67 (**K, O**) or with the neuroendocrine label PGP 9.5 (**L**). The scale bars in the left column represents 100 µm and the scale bars in the right 3 columns represents 25 µm. Arrows indicate mitotic figures and blood vessels are labeled.

Orthotopic ultrasound guided xenografts faithfully recapitulated many of the histological hallmarks of neuroblastoma. Xenografts showed some heterogeneity in terms of cell size and immunohistochemical staining patterns (Figure 2), and the presence of cells with a more differentiated somewhat ganglion-like appearance in tumors from some but not all cell lines. Similar to the mouse tumors, all tumors of the xenografts tumors stained positive for the neuroendocrine marker PGP 9.5 (Figure 2). Mitotic figures were consistently seen (Figure 2) and proliferation, indicated by Ki67, was notably greater in areas around blood vessels and at the peripheral margins of the tumor (Figure 2). Regions of xenografts were also immunoreactive for synaptophysin, neuron specific enolase (NSE) and tyrosine hydroxylase (TH), however; these markers were not as ubiquitously expressed as PGP 9.5 (Figure 2).

Electron microscopy also demonstrated that the xenograft tumors resembled the human N-MYC amplified tumors as well as the murine TH-MYCN tumors. The human xenograft tumors contained areas enriched in dense core synaptic vesicles and synapses and junction typical of neuronal cells (Figure S2). There were also areas of lipids dispersed throughout the tumors.

**Figure 3:**
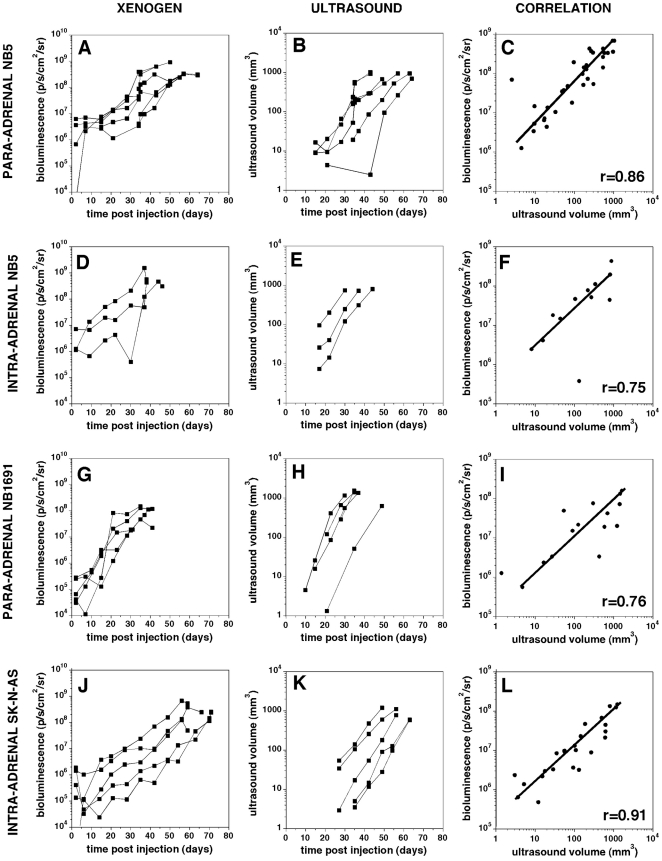
Growth of Orthotopic Neuroblastoma Xenografts. Bioluminescence **(left column)** and ultrasound volume **(center)** are plotted against time after injection (days post injection, dpi) for NB5 para-adrenal (**A–C**), NB5 intra-adrenal xenografts (**D–F**) and for NB1691 (**G–I**) and SKNAS (**J–L**) para-adrenal xenografts. A correlation analysis between the bioluminescence (y-axis) and ultrasound volume (x-axis is presented for the various xenografts in the right column. The correlation co-efficient is provided in the lower right hand of panels **C, F, I** and **L**.

Tumor growth was monitored by sequential Xenogen imaging. Growth curves generated from this data are shown in Figure 3. Paradrenal xenografts established from cell line NB1691 showed the most aggressive growth rates reaching a tumor volume of 600 mm^3^ after 31.8+/−8.8 days (n = 5). NB5 paradrenal xenografts grew slightly slower, reaching a volume of 600 mm^3^ after 52.2+/−9.8 days (n = 6) when transplanted into the retroperitoneal space near the adrenal or 36+/−3.5 days (n = 3) when injected directly into the adrenal gland. Only 20% (3/15) of paradrenal SKNSH xenografts reached a volume of 600 mm^3^ and this took on average 103.3+/−23.4 days (n = 3) (Figure S4). The remainder of SKNSH xenografts either failed to engraft (based on permanent loss of the Xenogen signal that was present immediately after injection) or regressed (initially signal was maintained or increased slightly during first few weeks and then gradually disappeared). Similarly, NB7 xenografts demonstrated inconsistent engraftment and growth with only 2/19 mouse tumors reaching a volume of 600 mm^3^, one after 43 days and the other after 142 days (Figure S4). The remaining NB7 xenografts either failed to engraft or remained as steady disease (maintained bioluminescent signal without evidence of growth) throughout the study. Thus, overall, xenografts from NB1691, NB5, and SKNAS provide consistent engraftment and growth rates whereas, xenografts from NB7 and SKNSH are less reliable.

### Diagnostic imaging of neuroblastomas in mice

**Figure 4:**
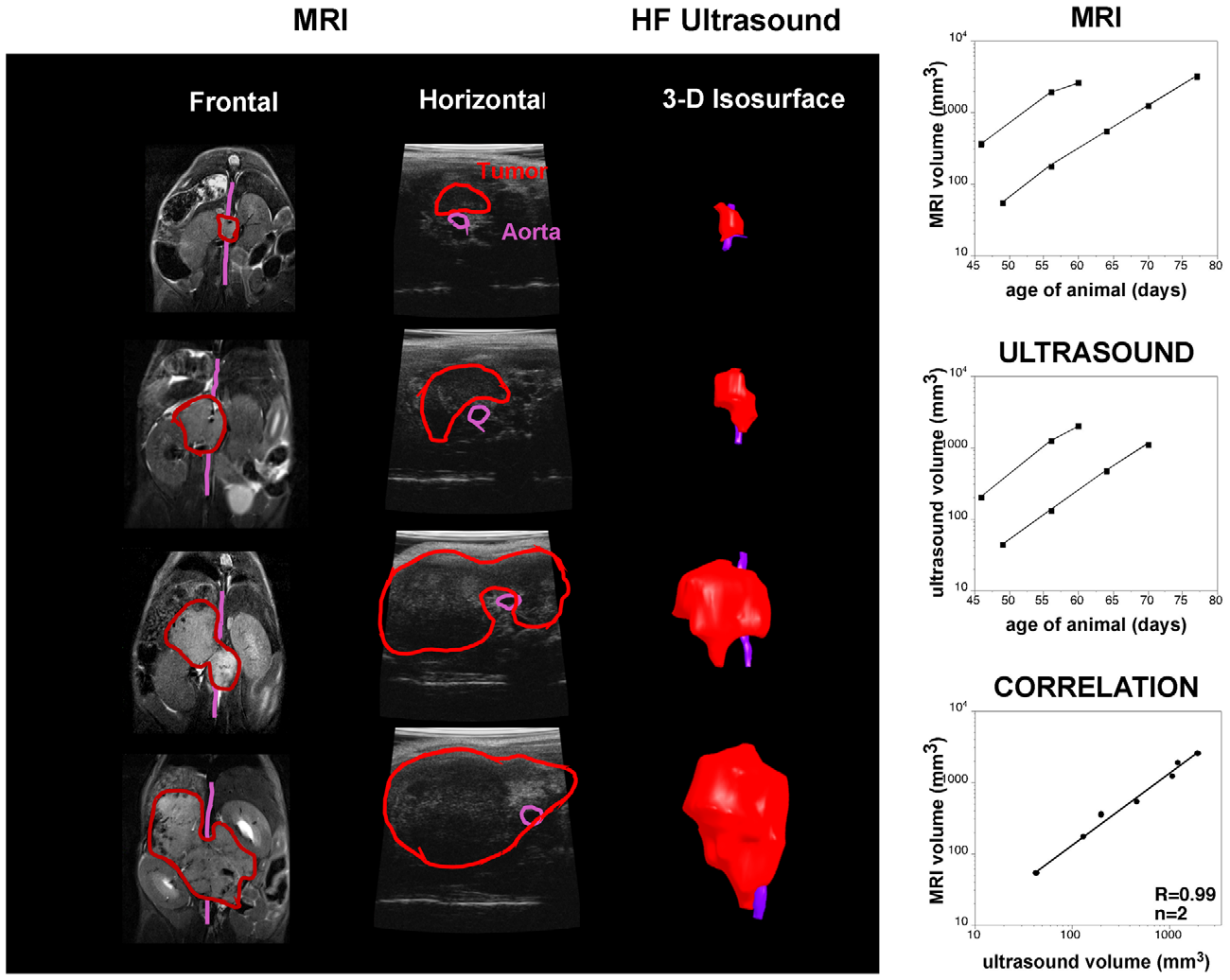
TH-MYCN mouse tumor imaging by MRI and ultrasound. The left column shows tumor progression at four distinct time points (top: first, button: last) as depicted by T2 weighted MRI. The tumor was manually segmented (red outline) on multi-slice coronal images and the volume averaged over all slices at each time point. The purple line traces the course of the aorta. Neuroblastomas are typically hypointense on T1-weighted images (VIBE data not shown) and hyperintense on T2-weighted images and can be seen on these images, especially in the later stages. The tumors appear to arise ventral to the aorta and demonstrate heterogeneous patterns, possibly from calcifications and hemorrhagic areas. Ultrasound data, also showing anatomical location of tumor (red) relative to aorta (purple), are presented as single slices (horizontal plane) and isosurface models generated from 3D data sets. Both imaging methods confirmed rapid growth of these representative tumors (and tight correlation across modalities) over the 30 day study period as described graphically to the right.

**Figure 5:**
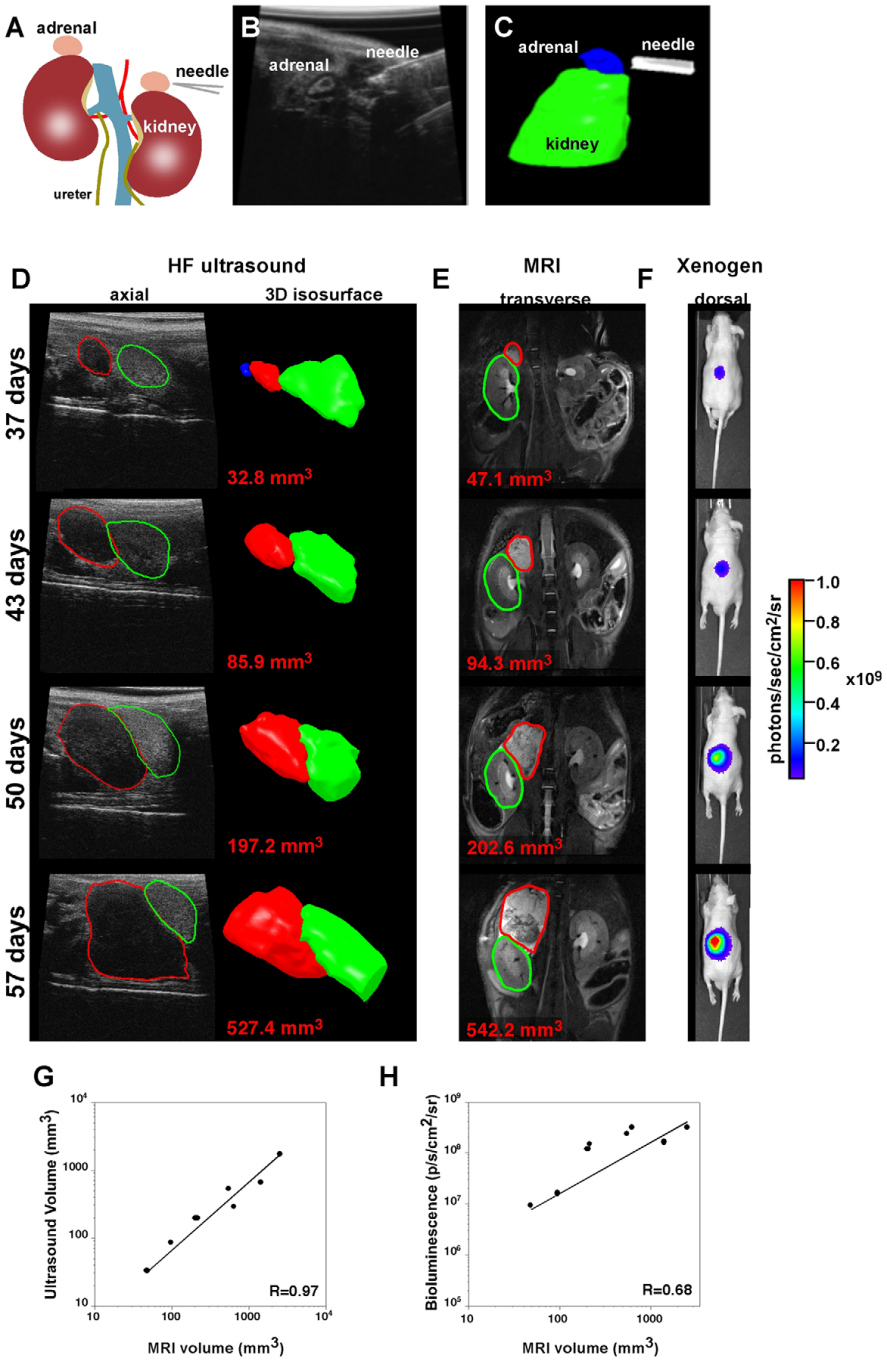
Xenograft neuroblastoma tumors were imaged by ultrasound and MRI. Orthotopic xenografts were produced by ultrasound guided needle injection of human NB cells to the para-adrenal or adrenal medulla in the mouse (**A–C**). Tumors were imaged by ultrasound, MRI and Xenogen imaging (**D–F**), tumor volumes determined and plotted (**G, H**). Ultrasound data (**C,D**) showing anatomical location of tumor (red) relative to the kidney (green) are presented as single slices (horizontal plane) and/or isosurface models generated from 3D data sets. MRI data are presented as single slices (frontal plane), again highlighting tumor relative to kidney. Bioluminescence images are presented with tumor highlighted colorimetrically (photons/second/cm^2^/steridian). Combination of the three imaging modalities clearly demonstrates efficient xenograft establishment and rapid growth from the para-adrenal space with appreciable correlation across the methods.

While the orthotopic xenograft studies had demonstrated the utility of Xenogen imaging for monitoring tumor growth, our preliminary studies indicated that the volume measurement remained linear when the tumors were relatively small but consistently underestimated the size of large tumors. Therefore, we tested the ability of ultrasound to accurately assess tumor volume in both model systems. For these studies, we imaged a cohort of 26 mice TH-MYCN hemizygous weekly or bi-weekly after initial detection of the tumors by ultrasound and assessed 13 mice with NB5 orthotopic xenografts. TH-MYCN mice were typically followed from week 5 through week 12 of age while the xenograft mice were followed beginning on day 2 after injection and then monitored weekly for 6–25 weeks. TH-MYCN tumors could be clearly detected by week 6–9 (Figure 4) while xenograft tumors were visible between weeks 4–6 (Figure 5). Ultrasound imaging proved to be an efficient and rapid method for screening large numbers of mice to identify initial tumors, determine their original location and for following the tumor growth and volume. Typically, 8–10 mice could be viewed in an hour time.

MRI on the other hand, provided superior detail and 3D volume measurements especially with large tumors, and allowed a more detailed diagnosis of the tumor origin and composition especially on T2 weighted imaging. This came, however, at the cost of about 25 min of table time per mouse.

**Figure 6:**
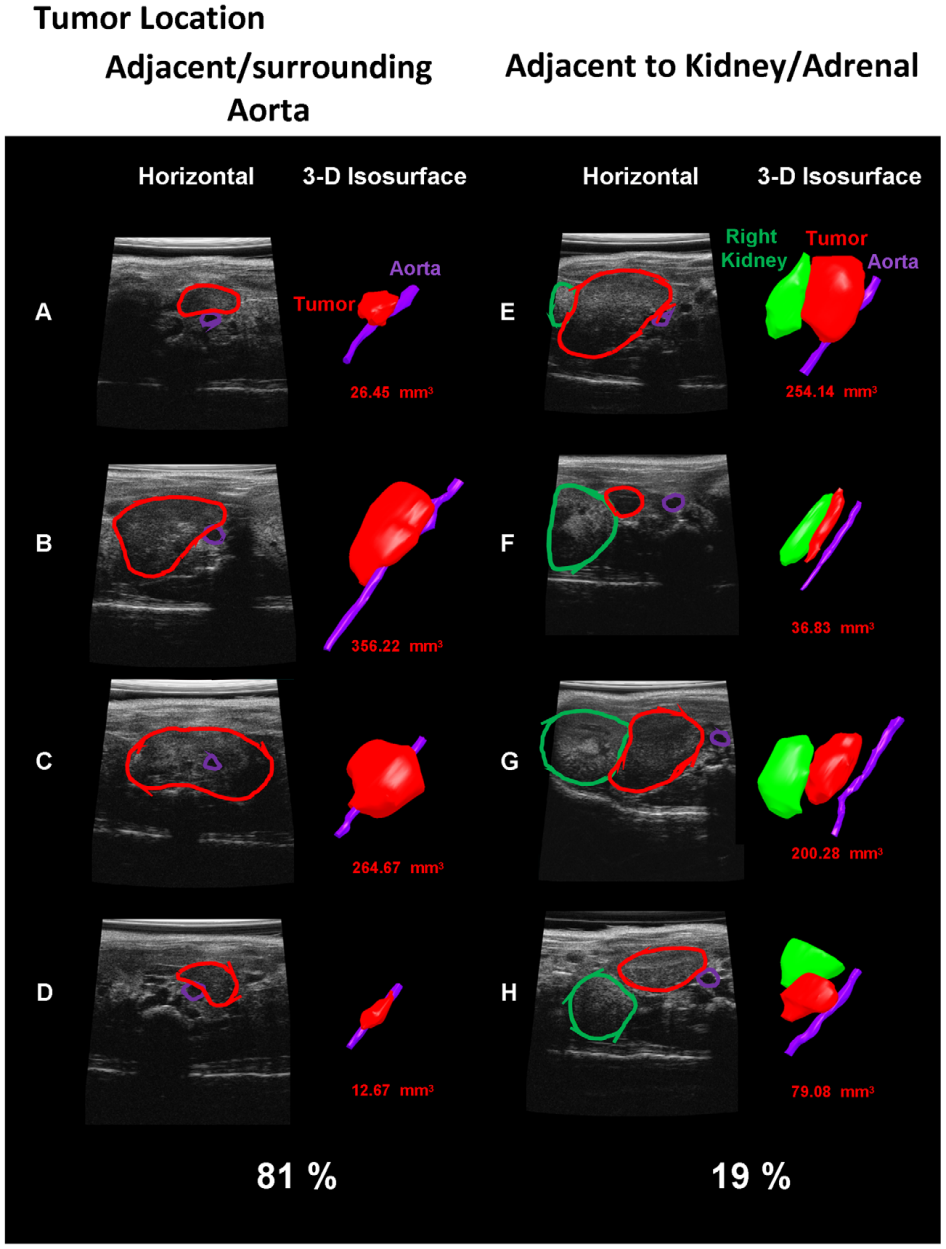
Location of TH-MYCN tumors as determined by ultrasound imaging. Ultrasound data showing the anatomical location of representative tumors (red) relative to aorta (purple) and right kidney (green). Data are presented as single slices (horizontal plane) and isosurface models generated from 3-D data sets which were also used to discern tumor volumes (mm^3^). Eighty-one percent of the TH-MYCN tumors (21/26) first appeared in the paravertebral ganglia adjacent to or surrounding the aorta of the mice, while the remaining 19% of the tumors (5/21) initially appeared closer to the adrenal or kidney.

The TH-MYCN tumors (21/26, 81%), first appeared very near or surrounding the aorta in the paravertebral ganglia of the mice, while the remaining 19% of the tumors (5/21) initially appeared closer to the adrenal or kidney by both MRI and ultrasound (Figure 6). All of the xenograft tumors appeared either in the para-adrenal region or within the adrenal depending on the site of injection. Examples of bioluminescence, ultrasound and MRI imaging of a developing xenograft tumor are shown in Figure 5. Importantly, both TH-MYCN and xenograft tumor volumes showed good agreement between the imaging methodologies (Figures 4 and 5).

### Spontaneous tumor regression in TH-MYCN mice

**Figure 7:**
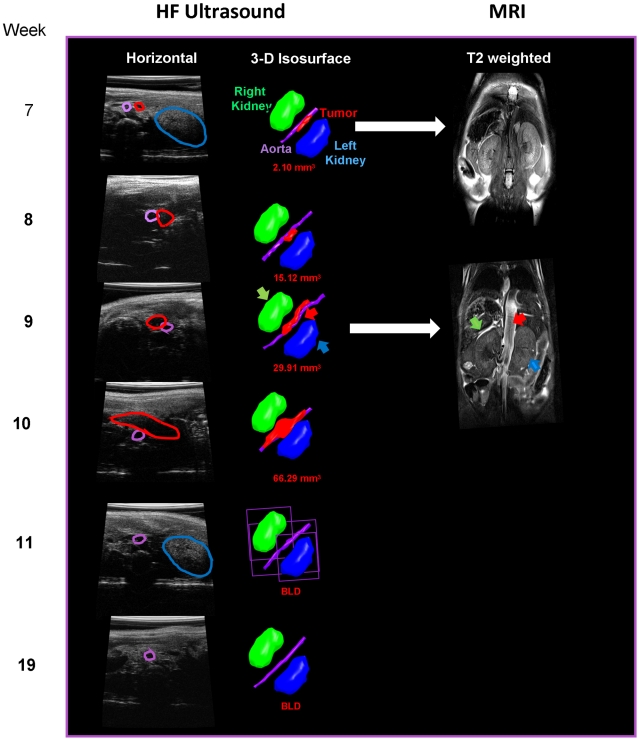
Monitoring of regressing TH-MYCN tumors by imaging. Regressing TH-MYCN tumors were imaged by ultrasound and MRI between weeks 8–10 and tumor volumes were determined. Ultrasound data **(left panels)** showing anatomical location of tumor (red), aorta (purple), right kidney (green) and left kidney (blue). Data are presented as single slices (horizontal plane) and/or isosurface models generated from 3D data sets **(middle panels)**. MRI data are presented as T2 weighted single slices (frontal plane), again highlighting tumor relative to kidney **(right panels)**.

**Figure 8:**
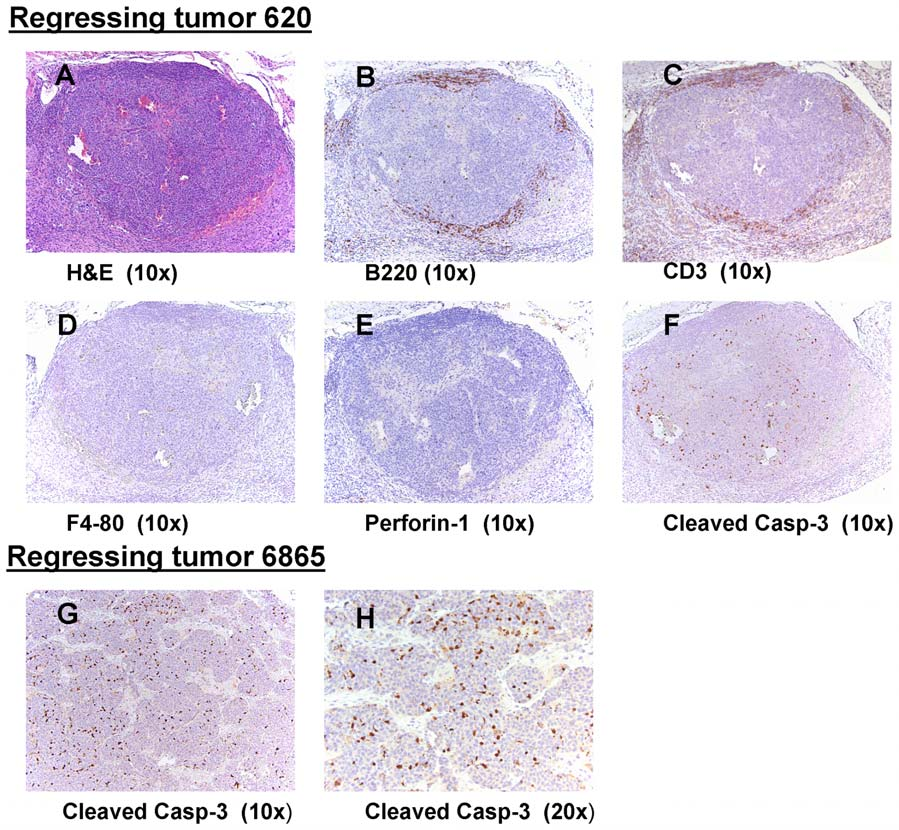
Morphology and immunohistochemistry of regressing TH-MYCN tumors. (**A**), Regressing tumor 620 stained with H&E, B-cell marker, B220 (**B**), T-Cell marker, CD3 (**C**), macrophage cells marker F4-80 (**D**), a CD8 T-cells and natural killer cells marker Perforin-1 (**E**) and cleaved caspase-3 (**F**). Regressing tumor 6865 stained with cleaved caspase-3 (**G, H**).

Interestingly, we noticed that 10% of the TH-MYCN tumors (5 out of 50 tumors total) exhibited a clear arrest in the growth between weeks 8–10 which lasted approximately 2 weeks and was followed by complete regression of the tumors based on ultrasound and MRI imaging (Figure 7). Hematoxylin and Eosin staining of the regressed tumors revealed that the cellular morphology of these tumors was similar to the TH-MYCN tumors that did not regress (Figure 8A). Infiltration of B and T cells was clearly observed near the peripheral edges of the tumors by staining with antibodies B220 and CD3 (Figure 8B,C). The regressed tumors also had low staining for F4-80, a marker for macrophages (Figure 8D). Only one of the regressing tumors (#6865) exhibited a significant increase in the percentage of cells that were undergoing apoptosis as judged by the presence of activated caspase-3 (Figure 8G,H compared to 8F). Tumor regression was also observed in some of the NB7 and SKNSH xenografts as described above and in Figure S4. Tumors typically regressed before they reached a volume of 100 mm^3^ in both models.

### Induction Chemotherapy in Neuroblastoma Orthotopic Xenografts

**Figure 9:**
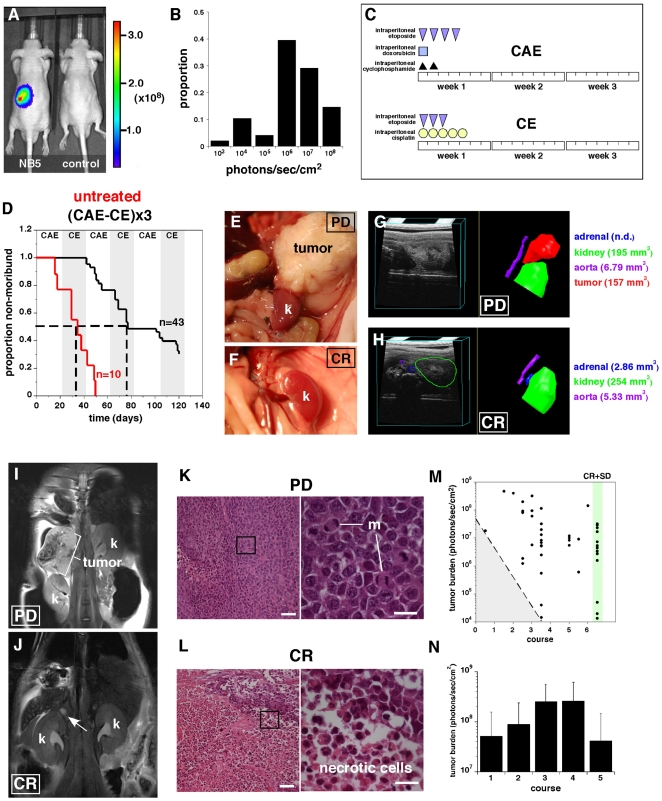
Induction chemotherapy in neuroblastoma. (**A**) Representative Xenogen image of an NB5-Luc orthotopic xenograft and uninjected control at enrollment. Scale is photons/sec/cm^2^. (**B**) Histogram of proportion of the 43 animals shown by tumor burden as measured by Xenogen. (**C**) Schedule of the etoposide, doxorubicin, cyclophosphamide (CAE) and the etoposide, cisplatin (CE) courses of chemotherapy. (**D**) Survival curve for 43 mice treated with alternating courses of CAE and CE for 18 weeks (6 courses) and 10 untreated control animals. The tumor burden was monitored by Xenogen and ultrasound. 30% of the mice survived therapy while all of the untreated mice reached moribund status within 50 days (**E,F**) Photograph of kidney (k) and para-adrenal space of a representative animal with progressive disease (PD) and complete response (CR). (**G,H**) Representative 3D ultrasound and tracing with volume measurements for a representative PD and CR showing volumes for adrenal (blue), kidney (green), aorta (purple) and tumor (red). (**I, J**) Examples of MRI of an animal with PD showing displacement of the kidney from the tumor growth (I) and a CR animal after completion of treatment showing normal kidney adrenal positions (arrow in J) where the tumor was previously located. (**K,L**) H&E staining of tumors from mice with CR and PD. Arrow indicates region of necrosis. (**M**) Plot of tumor burden at diagnosis as measured by bioluminescence (xenogen and the course of chemotherapy when the animal became moribund. The gray shaded region with dashed line indicates the minimum burden at each course where progression could be detected. The group of CR and SD that completed 6 courses of therapy are highlighted in green. (**N**) Histogram of tumor burden just before each course of chemotherapy. Bars represent the mean and standard deviation of all surviving animals at each time point. Scale bars: K, L, = 50 µm.

The new ultrasound guided neuroblastoma xenograft approach combined with validation of diagnostic imaging to study tumor growth and response in vivo provides us with the opportunity to test new chemotherapeutic agents. To test the feasibility of preclinical testing in our orthotopic neuroblastoma xenograft model and establish a baseline response for current standard of care for neuroblastoma, we injected 200,000 NB5-Luc cells (NB5-Luc was generated by infection of the NB5 cells with luciferase expressing retrovirus as described in Materials and Methods) into the left para-adrenal space of nude mice. 2–3 weeks after injection, we monitored tumor engraftment and enrolled 43 mice on study that had localized bioluminescent signal (Figure 9A). The majority of mice (36/43) had signals of ≥10^6^ photons/sec/cm^2^ (Figure 9B). The animals received the same drugs that are routinely administered for induction chemotherapy to treat neuroblastoma patients. The first course was cyclophosphamide, doxorubicin and etoposide (CAE) and the second course was etoposide and cisplatin (CE) (see Materials and Methods for detailed description of dosing and toxicity) (Figure 9C). Alternating CAE/CE treatments were repeated for a total of 6 courses over 18 weeks. Before and after each course, the tumor burden was monitored by bioluminescence and ultrasound as described above. Overall, 13/43 (30%) of the mice treated with induction chemotherapy completed 6 courses of therapy while all of the untreated mice had progressive disease and were euthanized by 50 days after being enrolled on study (Figure 9D). Among those 13 animals that completed therapy, 9 were complete response (CR) as indicated by the absence of bioluminescence, ultrasound signal (Figure 9G,H), MRI signal (Figure 8 I,J) and lack of visible tumor at necropsy (Figure 9E,F). The remaining 4 animals were classified as stable disease and showed extensive necrosis in histopathological analysis (Figure 9K,L).

To determine if there was any correlation between tumor size at diagnosis and outcome, we plotted the initial tumor burden (bioluminescence) versus the stage when their tumor progressed and they became moribund (Figure 9M). During the first 3 courses (9 weeks) of therapy, there was a clear relationship between initial tumor burden and progression (shaded region in Figure 9M). However, after the 3^rd^ course, there was no correlation between initial tumor burden and response to this standard of care chemotherapy regimen. Moreover, those animals that showed a complete response or stable disease after 6 courses of therapy had an initial tumor burden of less than 3×10^7^ cells. Therefore, for future studies, we recommend enrolling animals with an initial tumor burden for the NB5-Luc cell line between 1×10^4^ and 3×10^7^ cells. To determine if 6 courses of therapy was essential for these studies to identify those animals that are likely to respond and those that are likely to progress, we plotted the tumor burden (bioluminescence) of all animals just before each course (Figure 9N). These data show a trend toward increased tumor burden over the first 4 courses but by the 5^th^ course, those animals with stable disease or complete response can be clearly identified. Taken together, these data establish the feasibility of performing preclinical testing of novel combinations of chemotherapy and provide a benchmark for the widely used standard of care induction chemotherapy for neuroblastoma.

## Discussion

The studies in this report reveal similarities and differences between the two mouse tumor model systems. At the cellular level the two models were highly similar in terms of appearance, immunohistochemical staining patterns and the presence of cellular junctions and synaptic dense core vesicles. Tumors from both models also contained a high percentage of proliferating cells characterized by both Ki67 staining and a large number of mitotic cells and exhibited regions with substantial numbers of necrotic and apoptotic cells. The similarities between the mouse and human tumors are also highlighted by the gene expression microarray studies which found a 68.1% Pearson correlation in quartiled and grade adjusted gene expression. However, there are also several differences including: the presence of large non-dividing ganglion-like cells in all the mouse tumors and larger, somewhat ganglion-like cells in only some of the human xenografts (xenograft SKNAS, for example, did not contain ganglion-like cells). In addition, the majority of the mouse tumors fail to macrometastasize beyond the local area. Eighteen genes were also differentially expressed (greater than one quartile difference) in the human and mouse tumors. These differences may contribute to their tumorigenicity and/or metastatic abilities. For example the mouse tumors expressed higher levels of BRCA2, a known tumor suppressor gene in breast and ovarian cancer and involved in maintenance of genome stability, specifically the homologous recombination pathway for double-strand DNA repair [33], [34]. We do not yet know if any of these 18 genes account for the differences in disease progression between the human and mouse neuroblastomas but our ultrasound guided engraftment procedure is suited for testing these hypotheses.

Our ultrasound studies showed that 81% of initial tumors in the TH-MYCN mice are found surrounding or in the vicinity of the aorta in the paravertebral ganglia. The percentage of tumors in humans originating from the paraspinal ganglia is ∼60% [1], [4]. We speculate that this site in the mouse provides factors from the blood system and from the adjacent adrenal that can enhance tumor growth and progression. Surprisingly, none of the mouse NB tumors originated in the adrenal medulla itself as confirmed by ultrasonography, histology and electron microscopy. These results are in agreement with recent works observing initial tumor formation in the TH-MYCN mouse in early postnatal sympathetic ganglia and not in the adrenal [20], [35]. However, in a few cases we observed that the mouse tumors invaded the adrenal at later stages of tumor progression. This is in contrast to the human disease where approximately 40% of the patient tumors originate in the adrenal medulla [1], [4].

Recording the *in vivo* regression of a small group of TH-MYCN tumors by ultrasound and MRI opens the possibility of using this model to study this process and suggests these tumors may exhibit similarities to human stage 4S tumor which also spontaneously regress. Understanding the regression process *in vivo* could help identify the biological pathways that will facilitate tumor growth arrest and drive the tumor to complete regression.

The standard of care protocol described in this paper will be useful for testing new drug compounds, alone or in combination with the standard CAE/CE treatment, for their efficacy in treating neuroblastoma. Our study suggests that the most accurate data will be obtained from combination of both mouse models in association with multiple imaging modalities since the TH-MYCN system allows testing of the drugs in an immune proficient model while the orthotopic xenograft system uses human cells grown in the proper microenvironment. The ultrasound guided injection technique can also be used to generate a new bank of neuroblastoma patient xenografts by injecting cells directly from the patient tumors into mice and subsequently passaging the tumors without ever growing the cells in culture. This xenograft bank would facilitate future drug testing and could potentially lead to treatments that are more tailored to individual patients.

## Materials and Methods

### Animals

TH-MYCN hemizygote mouse were purchased from NCI mouse repository (strain # 01XD2) on genetic background 129X1/SvJ and kept on this genetic mouse background. CD-1 nude immunodefecient mice were purchased from Charles River (strain code 087, heterozygous).

This study was carried out in strict accordance with the recommendations in the Guide to Care and Use of Laboratory Animals of the National Institute of Health. The protocol was approved by the Institutional Animal Care and Use Committee at St. Jude Children's Research Hospital (IACUC protocols 420 and 393). All efforts were made to minimize suffering. All mice were bred and housed in accordance with approved IACUC protocols. Animals were housed on a 12-12 light cycle (light on 6am off 6pm) and provided food and water *ad libitum*.

### Generation of Bioluminescent Cell Lines

NB cell lines NB5, NB7, NB1691 [23], [31], [32], SKNAS (ATCC CRL-2137), and SKNSH (ATCC HTB-11) were infected with a MSCV-luciferase-IRES-zeocin retrovirus. Zeocin selection was initiated 48 hours after infection as described previously [36] (Institution Biohazard Committee approval 02-152). After selection the cell lines were screened to verify luciferase activity. Cells were maintained in RPMI-1640 medium with 10% fetal calf serum.

### Immunohistochemistry and electron microscopy of the tumors

Paraffin-embedded formalin-fixed tumors were immunostained for histochemical analysis with the following antibodies: B220 (CD45R/B220) (B.D. Biosciences, CA, USA, cat. 553084) (dilution 1∶10,000), Caspase-3 (BioCare Medical, CA, USA, cat. CP229C) (dilution 1∶100), CD3 (Santa Cruz, CA, USA, cat. sc-1127) dilution 1∶350), Chromogranin (Immunostar, WI, USA, cat. 20085) dilution 1∶5,000), F4/80 (Caltag, CA, USA, cat. MS48000) dilution 1∶1,000), Ki67 (ThermoShandon, CA, USA, cat. RM-9106) (dilution 1∶200), MAP-2 (Millipore, CA, USA, cat. AB5622) (dilution1∶250), NFP (DAKO, CA,USA, cat. MO762) (dilution 1∶40), NSE (DAKO, CA, USA, cat. MO873) (dilution 1∶50), Perforin (Abcam, MA, USA, cat. ab16074) (dilution1∶4,000), PGP9.5 (Morphosystems, NC, USA, cat. 7863-0504) (dilution1∶10,000), Synaptophysin, (ThermoShandon, CA, USA, cat. RM-9111) (dilution1∶100) and TH (Vector, CA, USA, cat. VP-T489) (dilution 1∶40).

TH-MYCN, xenograft and human patient tumors were fixed for electron microscopy in 4% glutaraldehyde in 0.1 Msodium cacodylate buffer pH 7.4 with 5% sucrose and post fixed in 0.2% osmium tetroxide in 0.1 M sodium cacodylate buffer with 0.3% potassium ferrocyanide for 2 hours. After rinsing in same buffer the tissue was dehydrated through a series of graded ethanol to propylene oxide, infiltrated and embedded in epoxy resin and polymerized at 70°C overnight. Semithin sections (0.5 micron) were stained with toluidine blue for light microscope examination. Ultrathin sections (70 nm) were cut and stained with Reynolds lead citrate. Examinations were made with a FEI Tecnai F20 200 Kv electron microscope with an AMT V600 digital camera. The use of human patient samples was approved by the institutional review board of St. Jude Children's' Research Hospital (XPD09-113) and all subjects provided written consent. The study was conducted in accordance with the Declaration of Helsinki.

### Ultrasound-Guided Para- or Intra-adrenal Xenografts

All ultrasound procedures were performed using the VEVO-770, fitted with a RMV-706 probe. Cells were suspended in Matrigel (BD Worldwide, Cat#354234) at a concentration of 2×10^4^ cells per microliter and placed on ice. Anesthetized recipient CD1 nude mice (isoflurane 1.5% in O_2_ delivered at 2 liters/min) were placed laterally on the imaging bed such that the left flank faced upward. In order to provide a channel for delivery of the implant, a 22 gauge catheter (BD Worldwide, Cat# 381423) was gently inserted through the skin and back muscle into the para-adrenal region and the hub was removed. A chilled Hamilton syringe fitted with a 27 gauge needle (1.25 inch) and loaded with 10 µl of the cell suspension was guided steretoactically through the catheter and positioned between the kidney and adrenal gland as visualized using ultrasound. The cells were injected into the region and the needle was left in place for 0.5–1 min in order to permit the matrigel component to set. The needle was then slowly removed, followed by gentle removal of the catheter. The same procedures were used for intra-adrenal injections although in these studies the needle was used to pierce the adrenal capsule and cells were deposited into the medulla of the adrenal gland.

### Bioluminescent Imaging and Quantification

For Xenogen imaging mice were given intraperitoneal injections of Firefly D-Luciferin (Caliper Life Sciences 3 mg/mouse). Bioluminescent images were taken five minutes later using the IVIS® 200 imaging system. The Living Image 3.2 software (Caliper Life Sciences) was used to generate a standard region of interest (ROI) encompassing the largest tumor at maximal bioluminescence signal. The identical ROI was used to determine the average radiance (photons/s/cm^2^/sr) for all xenografts from all time points. Biolumicescence and ultrasound volume per individual animal were plotted on a double Y axis using Kaleidagraph Software using the most appropriate scale for each cell line.

### Ultrasound imaging of the TH-MYCN mice and Xenografts

All ultrasound studies were performed using the VisualSonics VEVO-770 High Frequency Ultrasound system (VisualSonics, Toronto, Canada). Briefly, animals were anesthetized (isoflurane 1.5% in O_2_ delivered at 2 liters/min) and hair covering the area to be imaged removed by application of hair removal cream (Nair). For scanning, animals were placed prone on the imaging stage, ultrasound transmission gel (Parker Labs inc., USA) liberally applied and the relevant transducer (RMV-706 at 40 MHz) lowered stereotactically to the surface of the animal. Tumor (normally characterized by irregular regions of hypoechoicity) was identified relative to normal tissue landmarks and volumes were determined by acquisition of high resolution (50–100 µm in-plane) images with a step size of 100 µm providing a close to isotropic data set. Tumor perimeters were then traced at 0.5 mm intervals and the VisualSonics software used to render and calculate calibrated volumes.

Once luminescence was above 10^6^ photons/s/cm^2^/sr in the xenografted animals, imaging was perfomed weekly with the Visualsonic VIVO-770 ultrasound system fitted with the RMV-706 probe at a frequency of 40 MHz. The field of view was set 15×15 mm. Weekly three-dimensional scans of the tumors were reconstructed using the Visualsonics software. Tumor margins were traced and volume measurements were determined by the Visualsonics software. Bioluminescence and ultrasound volume per individual animal were plotted on a double Y axis using Kaleidagraph Software. Each cell line is represented with the best scale for that cell line.

### MRI imaging of the TH-MYCN mice and the Xenografts

Magnetic Resonance Imaging (MRI) was performed using a 7-Tesla Bruker Clinscan animal MRI scanner (Bruker BioSpin MRI GmbH, Germany) equipped with a Bruker BGA12S gradient (660 mT/m with 4570 T/m/s slew rate). Animals were anesthetized using isoflurane (1.5–3% in O_2_) for the duration of data acquisition. Briefly, animals were restrained on an MR safe bed (Bruker BioSpin MRI GmbH, model number T10211) in a prone head-in-first position. A 4 channel phased-array rat brain coil (Bruker) was used for signal reception. The coil was centered over the kidneys of the mice.

For Contrast Enhanced MR studies (CE-MRI), contrast agent (0.1 mmol/kg, Magnevist, Berlex, Montville, NJ), which corresponds to 50 µL of diluted contrast agent for a 25 g mouse, was injected through a tail-vein catheter [37], [38] and immediately flushed with 25 µL saline.

A stack of 25 coronal, T2 weighted, Turbo Spin Echo (TSE) [39] images (0.5 mm thickness) was acquired with no gap between the slices. The field of view (FOV) was 35×35 mm^2^ with a matrix size of 256×256. The echo time (T_E_) was 60 ms and the repetition time (T_R_) was 2610 ms. Receiver bandwidth (BW) was 235 Hz/pixel. 4 averages were used. For CE-MRI, VIBE [40] was used to rapidly acquire a T1 weighted 3D volume before and after injection. Orientation, matrix and FOV were identical to the T2 weighted images. 3D slab thickness was 14 mm with 56 slice encoding steps; the resulting voxel size was 0.14×0.14×0.25 mm^3^. Other sequence parameters were: TR = 10 ms, TE = 2 ms, flip angel = 15°. A partial Fourier factor of 6/8 was used in phase encoding, and slice selection direction. Each scan took 1.5 minutes and was repeated 12 times without pause to provide a time-course. Injection of contrast agent was performed immediately after the 4^th^ VIBE sequence. All images were read and processed on a Siemens console using Syngo MR B15 software (Siemens AG, Erlangen, Germany).

### RNA extraction and microarray expression analysis

RNA was extracted from TH-MYCN mouse tumors using Trizol reagent (Invitrogen) . RNA quality was confirmed by analysis on the Agilient 2100 Bioanalyzer. Total RNA (100 ng) was processed in the Hartwell Center microarray core according to the Affymetrix eukaryote two-cycle target assay (http://media.affymetrix.com/support/downloads/manuals/expression_analysis_manual.pdf). Biotin-labeled cRNA (10 ug) was hybridized overnight at 45°C to the Mouse Genome 430 2.0 GeneChip array which interrogates more than 39,000 transcripts. After staining and washing, arrays were scanned and expression values summarized using the MAS5 algorithm as implemented in the GCOS v1.4 software (Affymetrix, Santa Clara, CA). Signals were normalized for each array by scaling to a 2% trimmed mean of 500. Probe set annotations were obtained from the Affymetrix website (http://www.affymetrix.com/analysis/index.affx) [41]. All data is MIAME compliant and the raw data has been deposited in a MIAME compliant database (GEO Gene Expression Omnibus at NCBI). See Figure S3 for more details on the microarray analysis and methods. GEO number (GSE27516).

### Standardized treatment of the xenograft tumors

CD1-nude mice were injected with 10 µL of NB5-luciferase cells (2×10^4^/uL) into the para-adrenal space. These mice were then screened weekly by Xenogen and the bioluminescence was measured. The target bioluminescence signal for enrollment was 10^5^–10^8^ photons/sec/cm^2^ (median 6.51×10^6^, mean 5.65×10^7^). Once this signal was reached the animals were enrolled and started chemotherapy the following Monday.

The chemotherapeutic drug combinations and schedule used in this study was designed to mimic the most common treatments of neuroblastoma in human patients. These drug combinations have not been published previously in mice. Therefore, when possible, relevant drug dosages were determined using pharmacokinetic studies (specifically, area under the curve analysis) conducted previously on mice and humans to determine appropriate doses for the study. Once enrolled, the mice received 6 courses of alternating chemotherapy with each course lasting 21 days (18 weeks total). All drugs were administered systemically by intraperitoneal injection. The drugs given during courses 1, 3, and 5 included cisplatin 2 mg/kg (daily on days 1–5) and etoposide 5 mg/kg (daily on days 2–4). The drugs given during courses 2, 4, and 6 included cyclophosphamide 125 mg/kg (daily on days 1–2), doxorubicin 3.5 mg/kg (day 1) and etoposide 1.3 mg/kg (day 1) then 6 mg/kg (daily on days 2–4). Additionally, each mouse received 1 ml of 0.9% normal saline subcutaneously (daily on days 1–5) to help maintain hydration. The animals did not receive chemotherapy during days 6–21 during each course.

The health of the animals was monitored daily throughout the therapy. Standard complete blood counts with differential (CBC-Ds) were obtained prior to the initiation of each course of chemotherapy. For CBC-D collection, approximately 30 µL of blood was collected from the facial vein and diluted with standard amounts of EDTA. Samples were processed immediately using the FORCYTETM Hematology Analyzer (Oxford Scientific, Oxford, CT). Moribund status was defined as tumor burden greater than 20% body mass, at which point the animal was euthanized.

## Supporting Information

Figure S1 **Variable morphology was seen in the TH-MYCN tumors.** Most areas consist of solid cords (**A, B**), with area with a molding/pavement appearance (**C,D**). Ganglion-like pockets of cells were spread throughout the tumors (**E,F**). 40× magnification.
(TIF)

Figure S2 **Electron microscopy of a representative TH-MYCN tumor, orthotopic human NB tumors and a Stage 4 MYCN-amplified patient tumor.** A representative TH-MYCN tumor **(upper left)**, a human stage 4 patient tumor **(upper middle)** and xenograft tumors derived from neuroblastoma cell lines **SKNSH** **(upper right)**, NB1691 **(lower left)**, NB5 **(lower middle)** and SKNAS **(lower right)**. The general appearance of the tumors is shown in (**A**) dense core vesicle, junctions, lipids and processes (**B–D**).
(TIF)

Figure S3 **PCA analysis of human microarray data.** Human NB data were obtained from three GEO neuroblastoma studies. Each source is represented by a different dot color. The **left panel** shows the principal components (PC) analysis before correction for NB data source. Axes are: x = PC1 explains 13.70% of the variability of the data, y = PC2 explains 9.34% of the variability of the data and z = PC3 explains 6.86% of the variability of the data. The **right panel** shows the components (PC) analysis after correction for NB source. Axes are: x axis PC1 explains 13.10% of the variability of the data, y axis = PC2 explains 7.27% of the variability of the data and z axis = PC3 explains 5.76% of the variability of the data.
(PDF)

Figure S4 **Growth of additional orthotopic neuroblastoma xenografts.** Bioluminescence **(left column)** and ultrasound **(middle column)** plotted against time after injection (days post injection, dpi) for SKNSH (**A**) and NB7 (**B**). A correlation analysis between the bioluminescence (y-axis) and ultrasound volume (x-axis is presented for the various xenografts in the right column. The correlation co-efficient is provided in the **(upper corner of right column)**.
(TIF)

Table S1 **Data on the 424 unigenes that differ between mouse and human by at least one quartile.**
(XLS)

Table S2 **Data on the 125 unigenes of interest NB differ between mouse and human by at least two quartiles.**
(XLS)

Table S3 **Data on the 18 unigenes that exhibit at least two quartile differences but do not exhibit species differences in the adrenal.**
(XLS)
