# Supplementary figures and images for: Preclinical Models for Neuroblastoma: Establishing a Baseline for Treatment
Source: PLoS One. 2011 Apr 29;6(4):e19133. doi: 10.1371/journal.pone.0019133 (PMC3084749; doi:10.1371/journal.pone.0019133)

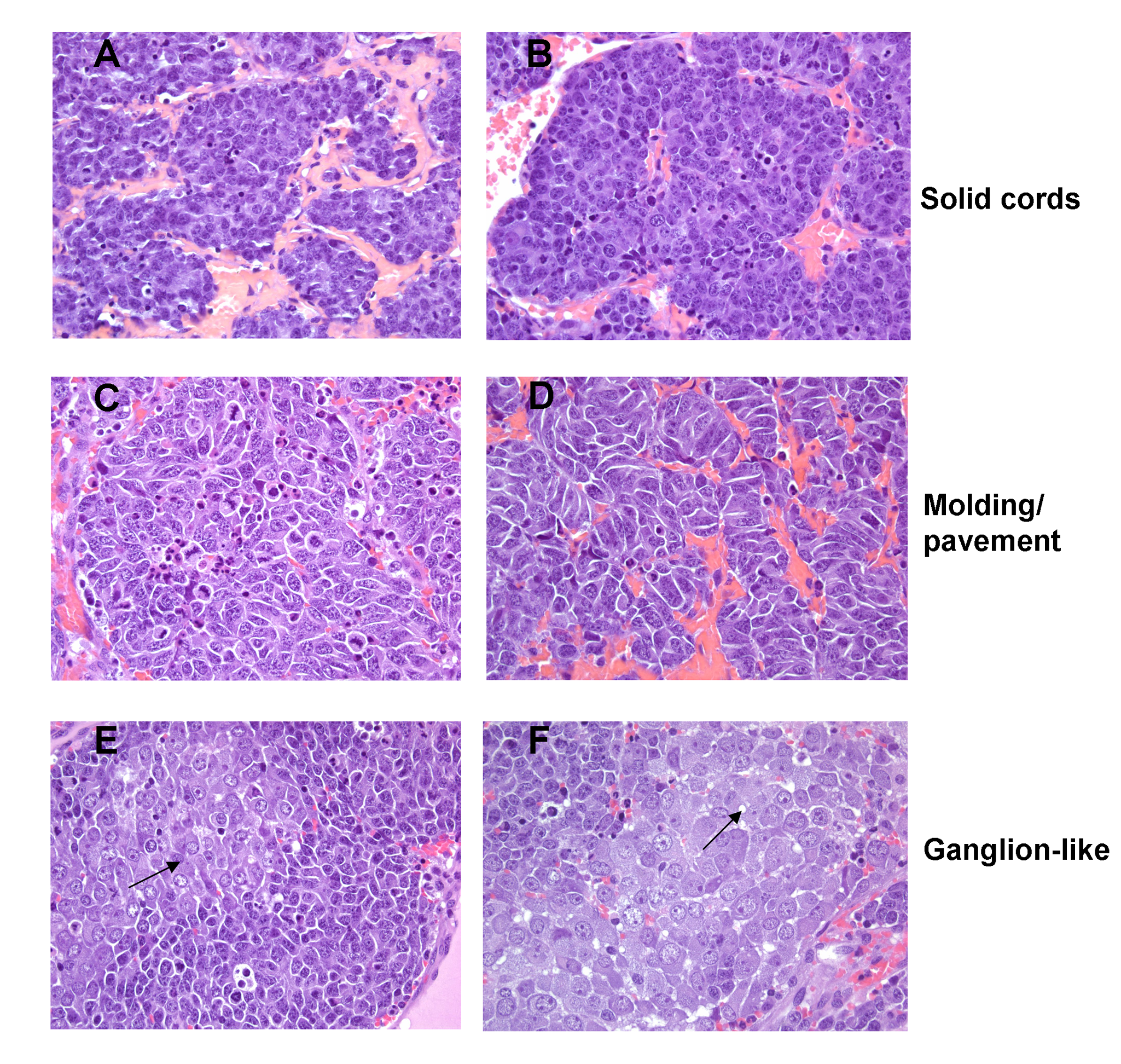

Supplement: Figure S1 — Variable morphology was seen in the TH-MYCN tumors. Most areas consist of solid cords (A, B), with area with a molding/pavement appearance (C,D). Ganglion-like pockets of cells were spread throughout the tumors (E,F). 40× magnification. (TIF) [file pone.0019133.s001.tif]

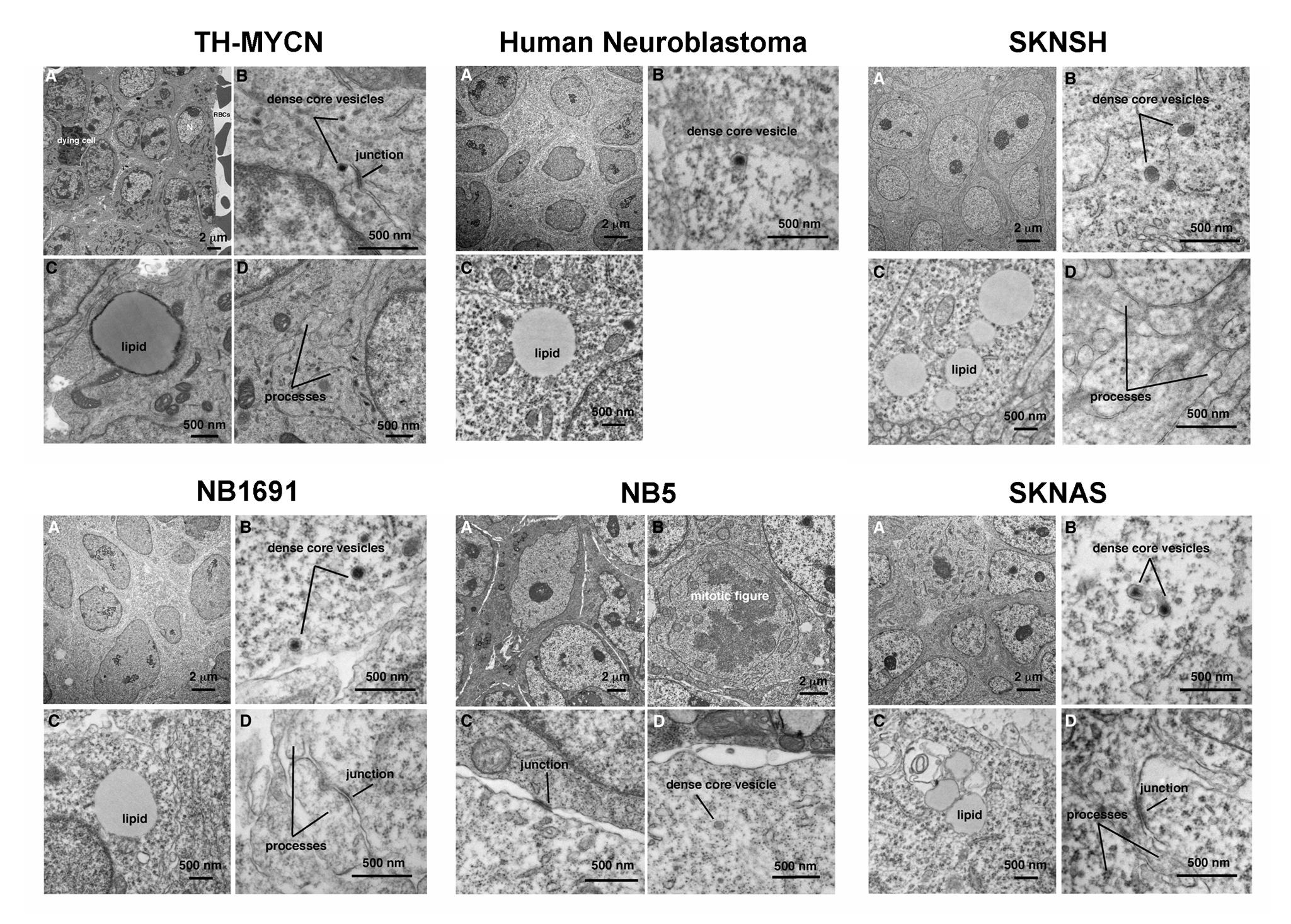

Supplement: Figure S2 — Electron microscopy of a representative TH-MYCN tumor, orthotopic human NB tumors and a Stage 4 MYCN-amplified patient tumor. A representative TH-MYCN tumor (upper left), a human stage 4 patient tumor (upper middle) and xenograft tumors derived from neuroblastoma cell lines SKNSH (upper right), NB1691 (lower left), NB5 (lower middle) and SKNAS (lower right). The general appearance of the tumors is shown in (A) dense core vesicle, junctions, lipids and processes (B–D). (TIF) [file pone.0019133.s002.tif]

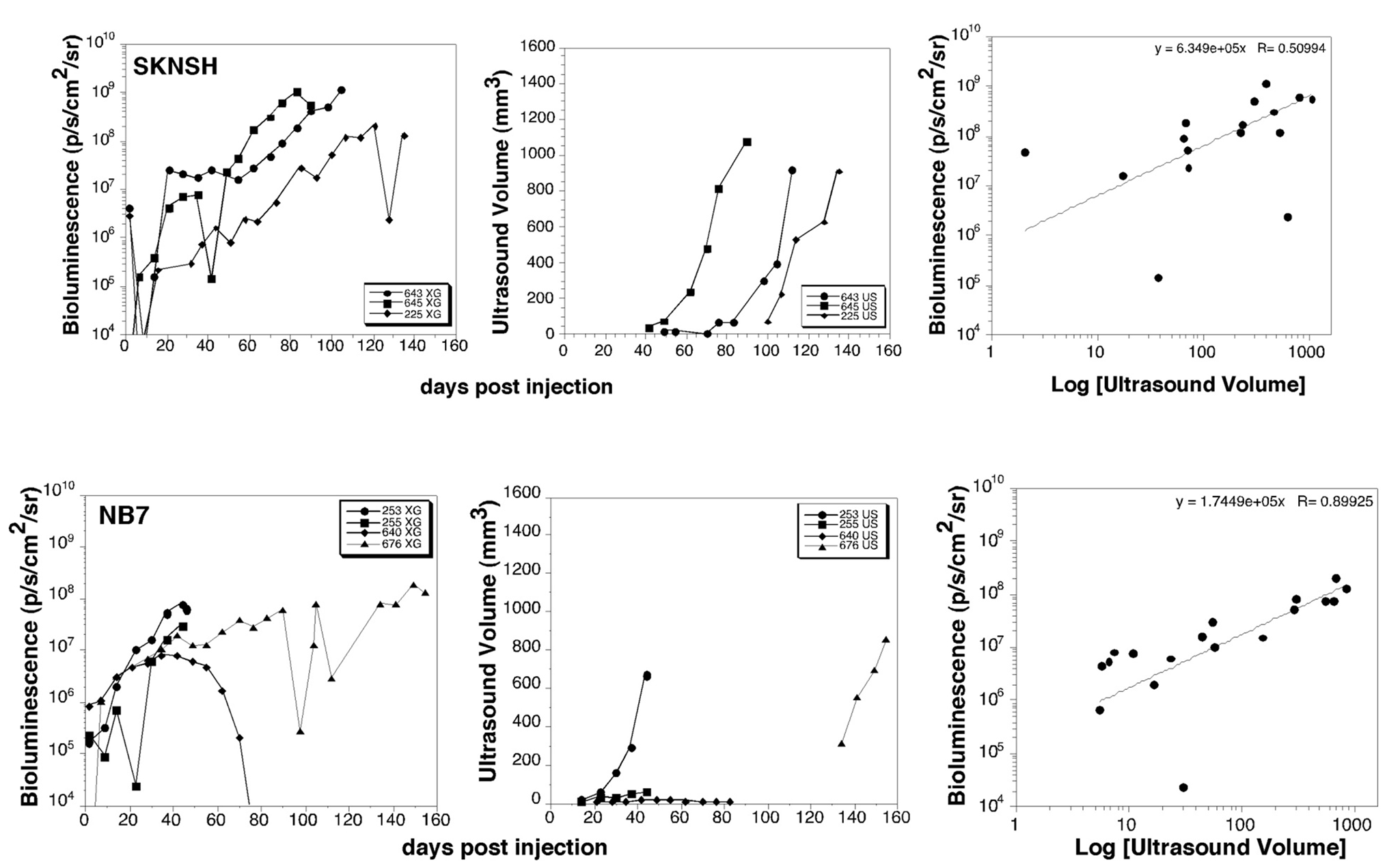

Supplement: Figure S4 — Growth of additional orthotopic neuroblastoma xenografts. Bioluminescence (left column) and ultrasound (middle column) plotted against time after injection (days post injection, dpi) for SKNSH (A) and NB7 (B). A correlation analysis between the bioluminescence (y-axis) and ultrasound volume (x-axis is presented for the various xenografts in the right column. The correlation co-efficient is provided in the (upper corner of right column). (TIF) [file pone.0019133.s004.tif]
