# Supplementary material for: Preclinical Models for Neuroblastoma: Establishing a Baseline for Treatment
Source: PLoS One. 2011 Apr 29;6(4):e19133. doi: 10.1371/journal.pone.0019133 (PMC3084749; doi:10.1371/journal.pone.0019133)
Supplement: Figure S3 — PCA analysis of human microarray data. Human NB data were obtained from three GEO neuroblastoma studies. Each source is represented by a different dot color. The left panel shows the principal components (PC) analysis before correction for NB data source. Axes are: x = PC1 explains 13.70% of the variability of the data, y = PC2 explains 9.34% of the variability of the data and z = PC3 explains 6.86% of the variability of the data. The right panel shows the components (PC) analysis after correction for NB source. Axes are: x axis PC1 explains 13.10% of the variability of the data, y axis = PC2 explains 7.27% of the variability of the data and z axis = PC3 explains 5.76% of the variability of the data. (PDF) [file pone.0019133.s003.pdf]

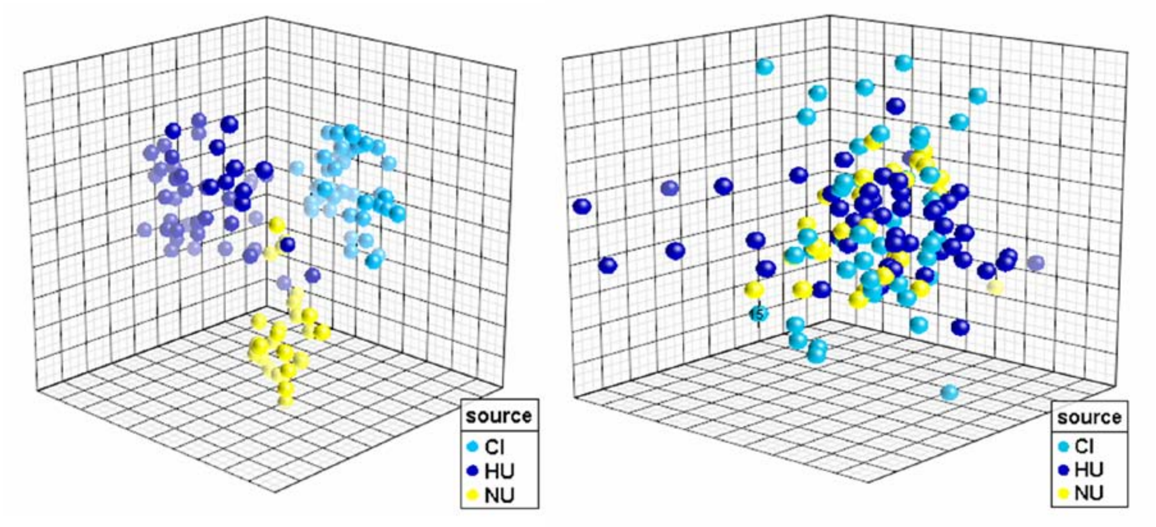

Human NB data were obtained from three GEO neuroblastoma studies (Gene Expression Ominibus-public data bases from the Curie Institute, Hiroshima University and Newcastle University and combined to give a total of 125 gene expression arrays. The data was then examined by principle component analysis (PCA) and collated with meta-data describing each tumor. The 125 arrays were adjusted by batch correction method included in Partek Genomics Suite 6.4. Data from Affymetrix human U133 v2 arrays and mouse Affymetrix 430 v2 arrays were processed using the RMA algorithm. This data was then used to examine data quality by PCA for each species separately. The corrections described above removed much of the data variability (compare right and left panels).

A. Source and number of human tumor microarray samples.

Source	Number of Tumors	Percent total
Curie Institute	45	36
Hiroshima University	50	40
Newcastle University	30	24
Total	125	100

References:

1. Anoueix-Lerosey I, Lequin D, Brugières L, Ribeiro A et al.(2008) Somatic and germline activating mutations of the ALK kinase receptor in neuroblastoma. Nature 16; 455(7215):967-70. PMID: 18923523 GSE12460

2. Lastowska M, Viprey V, Santibanez-Koref M, Wappler I et al. (2007) Identification of candidate genes involved in neuroblastoma progression by combining genomic and expression microarrays with survival data. Oncogene; 26(53):7432-44. PMID: 17533364 GSE13141.

3. Expression data of human neuroblastoma tissue samples. Unpublished GSE16237 Contact name Eiso Hiyama E-mail(s) eiso@hiroshima-u.ac.jp Hiroshima University

B. Human tumor frequency by stage and MYCN status

Stage	Myc amp +	Myc amp -	Total
1	4	29	33
2	2	15	17
3	4	9	13
4	4	21	24
4S	0	17	17

C. Tumor frequency stage and MYCN status by source

Stage	Curie Institute MYCN Amplification		Hiroshima University MYCN Amplification		New Castle University MYCN Amplification	
	+	-	+	-	+	-
1	3	5	1	20	0	4
2	1	8	1	5	0	2
3	3	4	1	4	0	1
4	7	6	4	8	10	10
**4S**	**-**	**8**	**-**	**6**	**-**	**3**
